# Influence of stabilising agents and pH on the size of SnO_2_ nanoparticles

**DOI:** 10.3762/bjnano.5.228

**Published:** 2014-11-20

**Authors:** Olga Rac, Patrycja Suchorska-Woźniak, Marta Fiedot, Helena Teterycz

**Affiliations:** 1 Faculty of Microsystem Electronics and Photonics, Wrocław University of Technology, Janiszewskiego 11/17, 50-372 Wroclaw, Poland

**Keywords:** nanoparticles, polyethylenimine, polyvinylpyrrolidone, surfactant, tin dioxide

## Abstract

According to recent research, the use of nanoparticles as a gas-sensitive material increases the selectivity and sensitivity and shortens the response time of a sensor. However, the synthesis of SnO_2_ nanoparticles presents many difficulties. The following article presents a simple and inexpensive method for the synthesis of SnO_2_ nanoparticles. The influence of the surfactant and polymer choice on the size of the resulting nanoparticles was investigated and a mechanism describing their interaction was proposed. It was found that stable colloids of SnO_2_ nanoparticles are formed in the presence of both PEI and Triton X-100 surfactants as stabilising agents. However, an additional factor essential for good stabilisation of the nanoparticles was an appropriate acidity level of the solution. Under optimal conditions, nanoparticles having an average diameter of about 10 nm are reproducibly formed.

## Introduction

Tin dioxide is an n-type semiconductor with a band gap width of 3.6 eV. It is characterised by good photocatalytic properties and in its presence, decomposition of an organic dye in the visible range of the electromagnetic spectrum may takes place [1]. Moreover, SnO_2_ is widely used for the production of transparent conductive layers or in the production of electrodes in lithium cells [2]. However, a SnO_2_ anode in a Li-ion cell has poor cycling stability [3]. A potential solution to this problem may be the use of nanoparticles. Tin dioxide nanoparticles of 3 nm diameter have a high charging capacity, however, this ability slightly diminishes after 60 cycles. It is expected that the SnO_2_ nanoparticles have the potential to replace conventional graphite anodes in lithium-ion cells [4].

In sensor research, many semiconducting metal oxides are used of which tin dioxide is the most widely studied and employed owing to its physicochemical properties. It features a high physicochemical stability and its electrical conductance changes predictably under the influence of various gases, which is the basis for operation of resistive gas sensors [5]. Despite the enormous number of studies devoted to chemical resistive sensors, there are still many problems that await solutions. For this reason, it is commonly understood that further progress in sensor development is dependent on (to a great extent) the research and development of new materials, and in particular, on nanomaterials due to their unique properties [6]. In the case of nanomaterials, the surface-to-volume ratio is very large, and the Debye λ_D_ length (a measure of the interaction area of a gas with the semiconductor surface) for most of the oxide semiconductor nanoparticles is comparable to the size of a wide range of dopants. For this reason, the electrical properties of oxide nanomaterials strongly depend on the kinetics of the processes occurring on its surface, which is extremely important during operation of chemical gas sensors [7–8].

Although the number of publications on the synthesis of oxide nanomaterials increases from year to year, the most often published studies are those presenting nanomaterials of various shapes and discussing their various properties [9]. Unfortunately, a mechanism for the formation of the nanoparticles is usually not proposed. This knowledge is, however, necessary to be able to reproducibly synthesize SnO_2_ nanoparticles, which would allow for their widespread use not only in lithium cells and gas sensors, but also in catalysis and other areas of science and technology.

Tin dioxide nanoparticles are typically obtained by simple vapour phase transport methods [10–11]. Nanocrystalline tin oxide for various applications is also frequently obtained by sol–gel methods [12–13], or by a chemical method in precipitation reactions from a cold solution [14]. The literature also describes a method for preparing tin dioxide nanowires with good sensor properties [15]. These nanowires were obtained by combining a spray pyrolysis method and annealing carried out under atmospheric pressure.

The subject of the synthesis of SnO_2_ nanoparticles in aqueous solution rarely occurs in the literature. SnO_2_ colloidal synthesis in cetyltrimethylammonium bromide (CTAB) micelles (where tin tetrachloride was used as a precursor and ammonia as precipitation agent) was presented by Wang et al. [16]. As a result of this reaction, an amorphous precipitate was obtained, dried and then annealed at 500 °C for 2 h. The average crystallite size of the resulting powder was 15–25 nm.

The precipitation reaction is one of the oldest preparation methods for various, poorly soluble, chemical compounds, including metal oxides. Currently, it is also one of the "bottom-up" preparation methods for colloidal systems of metal oxides. The advantage of this method lies undoubtedly in its simplicity, but also the fact that by controlling the basic reaction parameters the size (as well as the substructure) of the resulting nanoparticles can be modified. Examples of the reaction parameters which can be used to tune the size include: concentration of the reactants, type of precipitant, type and concentration of the stabilising agent, and temperature.

The main feature of colloidal systems in general (not only metal oxide colloids containing nanoparticles) is their tendency to reach the lowest thermodynamic energy state by the agglomeration of nanoparticles. This process usually results in an almost complete loss of the typical characteristic properties of nanomaterials, for example, the specific sensor or photocatalytic properties. For this reason, colloidal metal oxide nanoparticles (regardless of the synthesis method) require the use of a stabilising agent to fully maintain a high state of dispersion, which allows for the specific properties particular to nanomaterials. However, the properties of nanoparticles are determined not only by their dimensions, but also by their shape and surface morphology. For this reason, the synthesis of both inorganic and organic non-agglomerated nanoparticles of controlled size and shape is carried out in the presence of stabilising substances. Commonly, two types of stabilisers are used, namely, polymers and surfactants [17].

The synthesis of nanoparticles in the presence of a polymer is one of the preferred ways to prevent their agglomeration. Polymers used as stabilising agents significantly affect the size, shape and surface properties of nanomaterials synthesised in their presence [18]. In 1718, Helcher applied a natural polymer (starch) to stabilise gold nanoparticles [19]. Today, synthetic polymers are commonly used to stabilise different types of nanoparticles. There are many concepts that describe the interaction between polymers and nanoparticles, of which the most popular are two theories on the formation of nanoparticle–polymer composites. The first theory involves the in situ synthesis of nanoparticles as a result of precipitation reactions of metal oxides occurring in polymeric micelles [20]. The second theory, although less popular, describes the stabilisation of polymers by molecules that results from the polymerisation of monomers which occur in the presence of nanoparticles. In this article, synthetic polymers were used as stabilisers, therefore, the formation of the nanoparticles occurs in accordance with the first of these theories.

The article presents a very simple method for the synthesis of tin(IV) oxide nanoparticles involving precipitation from the aqueous solution containing the inorganic tin precursor, a polymer of appropriate composition, and a non-ionic surfactant. The influence of the individual reactants as well as the solution acidity on the dimensions of the resulting nanoparticles was analysed. The understanding of the interaction between these components of the solution is critical both for the synthesis of metal nanoparticles as well as metal oxides with semiconducting properties, including tin dioxide.

## Results and Discussion

### Analysis of received SnO_2_ nanoparticles (basic solution)

The aqueous solution in which the precipitation reaction for tin dioxide was carried out contained a tin(IV) chloride precursor, polyethylenimine (PEI) or polyvinylpyrrolidone (PVP), and *t*-octylphenoxypolyethoxyethanol (Triton X-100) as a surfactant with a hydrophilic–lipophilic balance (HBL) of 13.5. Anhydrous tin(IV) chloride is a strong Lewis acid and easily forms complexes [21]. In contrast, PEI is a synthetic cationic polymer which may be present in the form of molecules with different degrees of branching and molecular weight varying over a wide range. With regard to structure, in its linear form, it contains secondary amino groups, and in its branched form, primary, secondary and tertiary amino groups are present [22]. PEI is a polyelectrolyte that is protonated in acidic solutions, whereas at pH > 8, it exists in the free alkaline form [23]. PEI is often used as a colloidal metal nanoparticle and metal oxide stabiliser. Studies show that many metal nanoparticles (such as silver, gold, zinc oxide or tin dioxide) synthesised in solutions not containing PEI have much larger dimensions than those synthesised in the presence of PEI [24]. However, there are many factors affecting the mechanism and effectiveness of stabilisation, for example, the polymer type and the average molecular weight of the polymer (*M*_w_).

**Figure 1:**
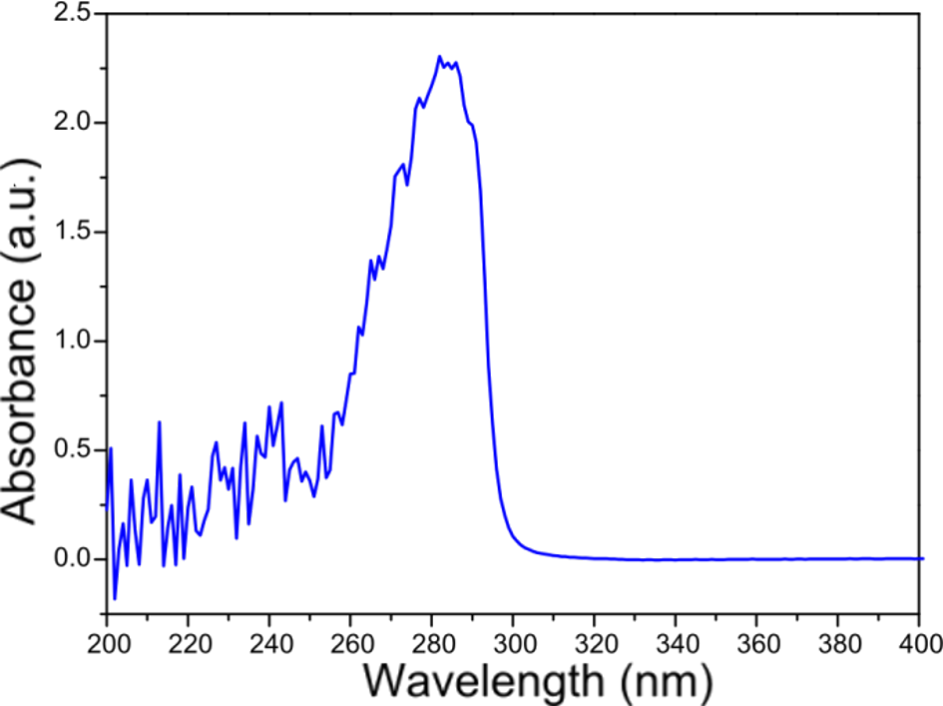
UV–vis spectrum of a tin dioxide colloid stabilised with PEI.

As a result of the precipitation reaction in a solution of standard composition (see Experimental section), a colloid was obtained. This was observed in the UV–vis spectrum of which a characteristic peak at a wavelength of 282 nm distinguishes the presence of tin dioxide particles in solution (Figure 1). The standard composition consists of: 8.24 mL of water, 3 mL of PEI *M*_w_ = 750 000 with a concentration of 1.0 mol/L, 6.56 mL of Triton X-100 with a concentration of 0.08 mol/L, 0.1 mL of SnCl_4_ with a concentration of 0.375 mol/L, and 0.21 mL of ammonium hydroxide with a concentration of 0.0714 mol/L.

**Figure 2:**
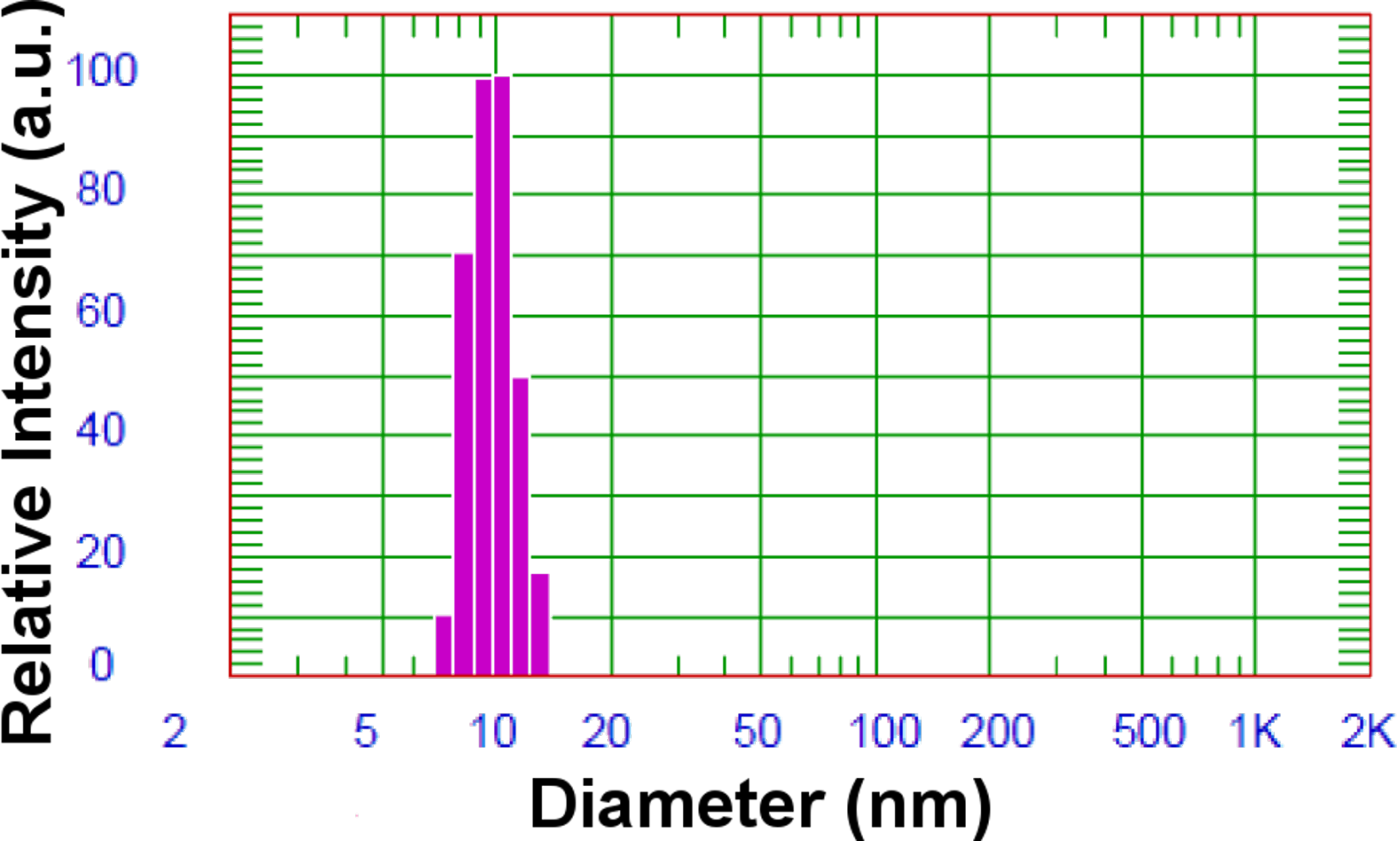
DLS results of the diameter distribution of the resulting nanoparticles formed in a solution with a standard composition.

Analysis of the dynamic light scattering (DLS) results revealed that in the solution of standard composition, nanoparticles of average diameter of approximately 8 nm are formed (Figure 2). For statistical analysis, a proprietary, high resolution, multimodal deconvolution analysis for proper measurement of the nanoparticle diameter was applied.

**Figure 3:**
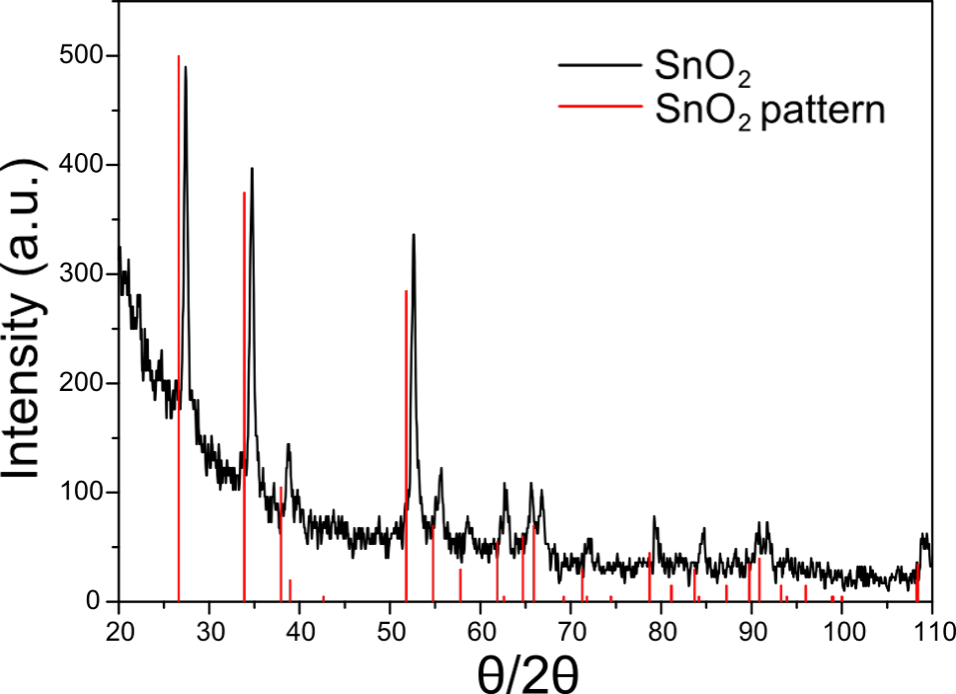
XRD pattern of prepared SnO_2_ nanoparticles.

X-ray diffraction (XRD) measurements indicate that the measured peaks for the nanoparticles correspond to a tetragonal phase of SnO_2_ (JCPDS #41-1445). There is not much difference between peak intensities corresponding to crystallographic planes (110), (101) and (211) and all are almost equally dominant (Figure 3). The average crystallite size of SnO_2_ nanoparticles was determined to be 16 ± 1 nm by the Scherrer equation, *D* = *K*λ/βcosθ, where *K* is the Scherrer constant (0.9), λ is the wavelength of the X-ray irradiation (1.5418 Å) and β is the width (FWHM) of the XRD peak obtained by fitting the peak data with the Gaussian function. The calculation of *D* for the three corresponding peaks at 27.4°, 37.6° and 52.6° was performed.

The average diameter of the nanoparticles as determined on the basis of XRD results was two times greater than those from DLS studies (Figure 2). This is due to differences in samples preparation for the measurements. The DLS method examines the intensity of scattered light passing through a solution of nanoparticles. In contrast, in the XRD method, the sample was dried and the stabilizing agents were removed, causing the agglomeration of nanoparticles and their recrystallization. However, nano-sized dimensions are still observed after heating the sample to 450 °C.

### The influence of surfactant on the average nanoparticle diameter

**Figure 4:**
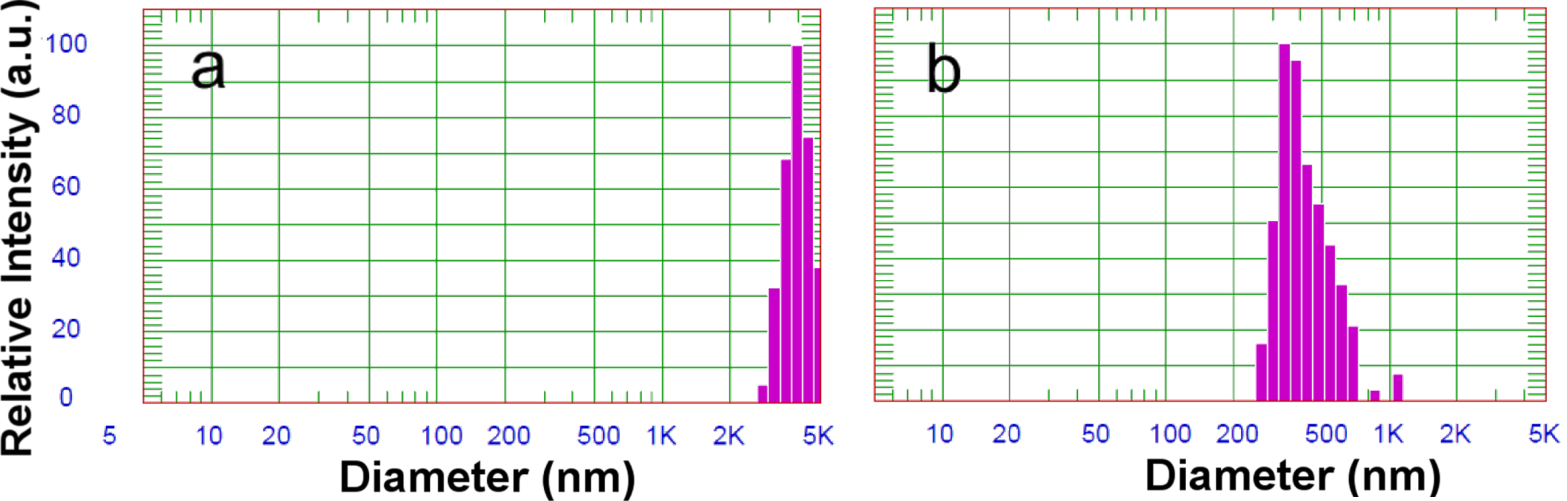
Size of SnO_2_ particles without the addition of (a) polyethylenimine, (b) surfactant.

In the first stage of the study, the use of two stabilising agents, namely, both a polymer (PEI) as well as a surfactant (Triton X-100) was investigated. In all of the solutions containing only one type of stabilising substance, large-sized particles were formed (Figure 4). This was especially true for the solution containing only the surfactant as the stabilising agent, where particles of a few micrometers (3.945 µm) in diameter were formed. In the case where only a polymeric stabilising agent was used the diameter was 395 nm. Thus, stable colloids of tin dioxide nanoparticles are formed only in solutions containing both stabilising materials (Figure 2).

**Figure 5:**
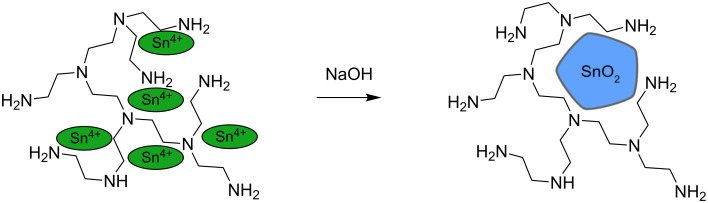
Schematic synthesis of polyethylenimine-stabilised SnO_2_ nanoparticles.

According to literature reports [25], in the first reaction step, PEI creates complexes with Sn^4+^ ions, and then the precipitated nanoparticles are surrounded by a polymer, resulting in a polymer matrix which is formed on its surface (Figure 5).

To facilitate this process, the corresponding change in the surface tension by the addition of a surfactant in the solution is required. To investigate the effect of surfactant concentration on the size of the nanoparticles, the samples were prepared with different amounts of Triton X-100 (the amount of PEI was held constant at 3 mL). Increasing the concentration of the surfactant in the basic solution causes a very distinct change in the average diameter of the nanoparticles (Table 1). In the case where the reaction mixture contained less than 8 mL of a surfactant solution, particles of nanometer dimensions were formed. After the addition of more surfactant, the diameter increased by about twenty times.

**Table 1:** Dimension of tin dioxide particles with varying amounts of surfactant. The amount of PEI was held constant.

Amount of Triton X-100 (mL)	Diameter (nm)

4.00	6
6.56	8
8.20	185
9.84	185

**Figure 6:**
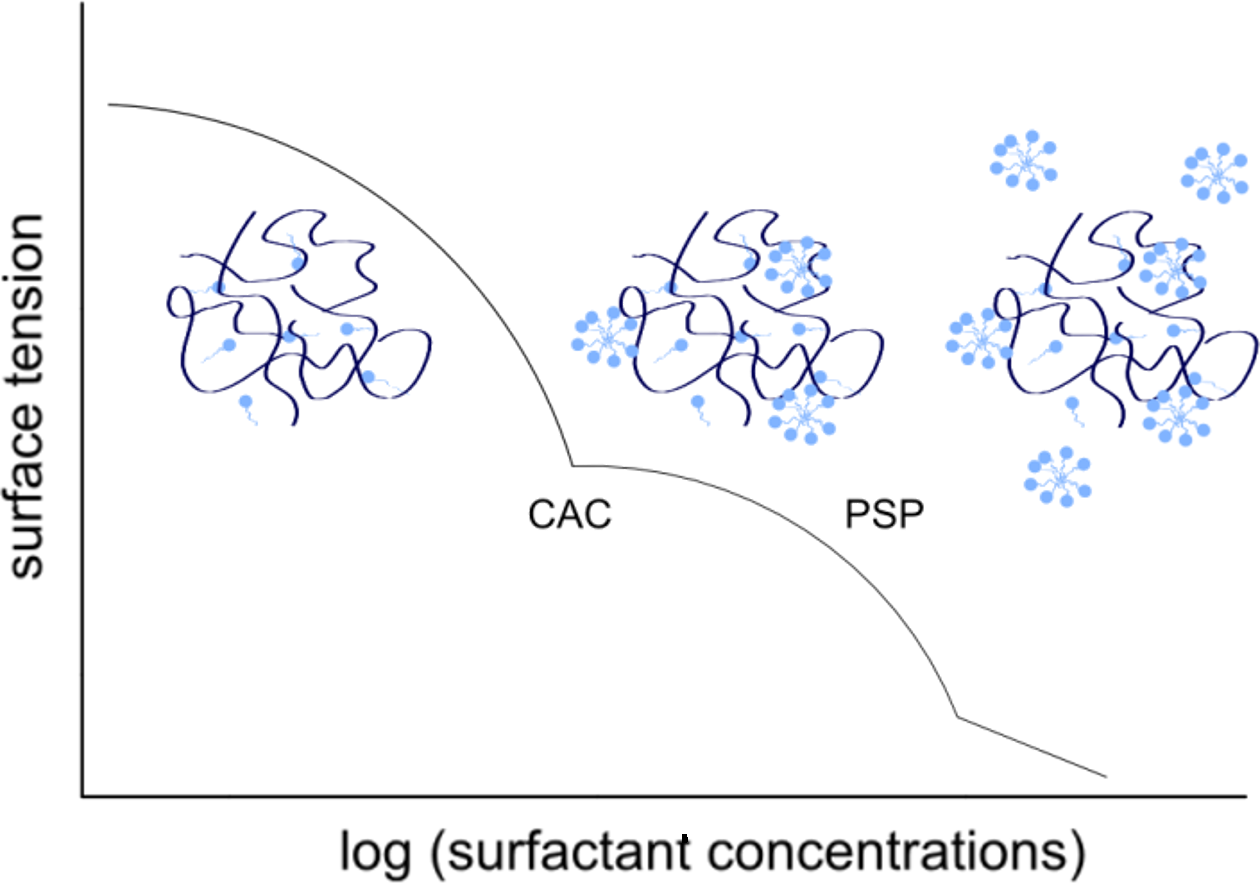
Scheme of possible interactions of PEI–Triton X-100.

Because the precipitation reaction was carried out in a solution containing both a polymer as well as a surfactant, it was necessary to take into account their mutual interaction. The studies used a non-ionic surfactant and a cationic polymer. According to the literature on this subject, when applying a small amount of the surfactant, the particles attach to the polymer chain and do not form micelles. In contrast, when the concentration is increased to the critical micelle concentration (CMC) point, the micelles are formed in the solution and bind to the active groups of the polymer. Upon further increase of the amount of Triton, the polymer saturation point (PSP) is reached. At this point, all of the active groups of the polymer are already occupied by individual particles or micelles from the surfactant and any excess forms free micelles, unrelated to the polymer chain (Figure 6) [26–27]. All of these processes affect the kinetics of the precipitation reaction of tin dioxide nanoparticles and cause a change in their size.

The analysis of the DLS results revealed that the surfactant is required during the precipitation reaction of SnO_2_, but also that the amount should not exceed a certain concentration. It is hypothesized that this is related to exceeding the PSP. Probably, after having exceeded this point, all active parts of the polymer are occupied by surfactant particles and then the polymer no longer serves as a stabiliser for the nanoparticles.

### The influence of polymer choice on the nanoparticle diameter

Two polymers, polyethylenimine (PEI) and polyvinylpyrrolidone (PVP), with three different average molar masses were used in the study. As demonstrated by the results of this study, in case of PVP, large tin dioxide particles approximately 1–2 µm in diameter were obtained (Table 2). The analysis of the results clearly showed that nanoparticles are formed only in the presence of high molecular weight, highly branched PEI. In the presence of low molecular weight PEI, microparticles of about 2 µm in diameter are formed. Many samples were made with different types of polymers (the amount of Triton X-100 was held constant at 6.56 mL).

**Table 2:** SnO_2_ particle size dependence on the type of polymer and its average molecular weight. The amount of Triton X-100 was held constant.

*M*_w_ of polymer	Size (nm)

Polyethylenimine, PEI	
2 000	2 000
25 000, branched	77
750 000	8
high molecular	12

Polyvinylpyrrolidone, PVP	
3 500	1 000
8 000	2 000
58 000	2 000

**Figure 7:**
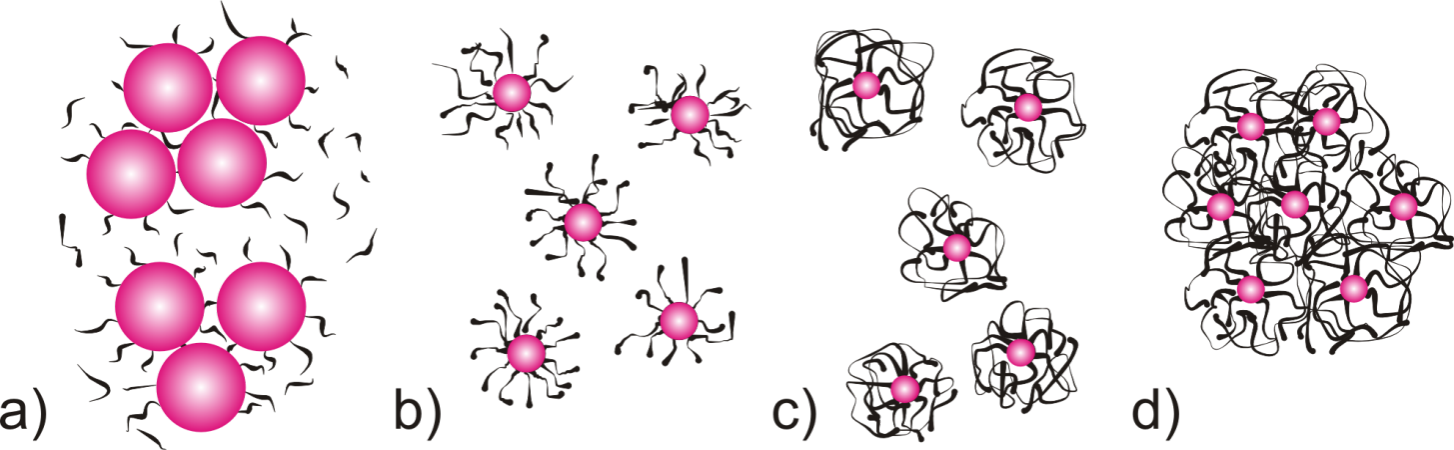
SnO_2_ nanoparticle stabilisation scheme depending on the chain length of PEI: a) low molecular weight, b) average molecular weight, c) high molecular weight and d) high molecular weight with too high concentration.

According to literature reports, the effective stabilisation of the nanoparticles occurs only in the presence of PEI of a suitable chain length and of appropriate structure [24]. According to the authors, one end of the polymer particles binds to the surface of the nanoparticles, while the remaining portion forms a loose layer around the nanoparticle. Thus, the double polymeric layer consists of the functional groups of the polymer chemically bonded to the surface of the nanoparticles and the polymeric chains form the outer layer. When the polymer particles are too short then they do not form an effective stabilising agent. For this reason, in the presence of low molecular weight PEI, large particles of tin dioxide are formed (Figure 7a). If a layer of high molecular weight or highly branched polymer surrounds the nanoparticles and extends into the solution, then it serves to protect the nanoparticles from agglomeration (Figure 7b,c).

Furthermore, research has shown that the size of tin dioxide nanoparticles depends not only on the average molecular weight of the stabilising polymer, but also on its concentration (Table 3). When 4.5 mL or less of PEI was added to the starting solution, the diameter of formed nanoparticles was about 10 nm. In solutions containing greater amounts of PEI, particles of ten times this diameter were formed.

**Table 3:** The dependence of the SnO_2_ particle diameter on the amount of PEI (*M*_w_ = 750 000). The amount of Triton X-100 was held constant.

Amount of high *M*_w_ PEI (mL)	Diameter (nm)

3.0	8
4.5	10
6.0	100
7.5	110
9.0	130

**Figure 8:**
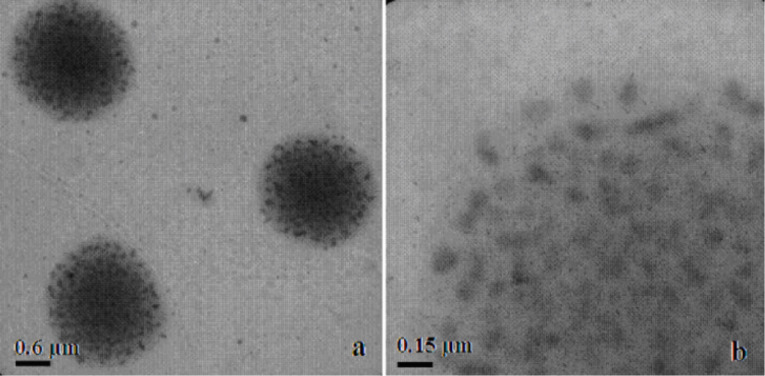
TEM images of agglomerated particles of SnO_2_ in different magnifications.

In order to demonstrate the agglomeration of the nanoparticles due to the addition of an excessive amount of polymer, TEM images of the sample containing 9 mL of PEI are presented in Figure 8. Moreover, the TEM images clearly show that the agglomerates which were created consisted of individual nanoparticles. This situation is demonstrated by the scheme presented in Figure 7d.

### The influence of pH on reaction kinetics

It is well known that tin oxide exhibits an amphoteric behaviour and that the stabilising agent PEI undergoes (to a varying extent) a protonation reaction depending on the acidity of the solution. For this reason, the impact of the concentration of the precipitating agent (i.e., ammonium hydroxide) on the dimensions of the formed nanoparticles was also tested. Studies have shown that in solutions containing more than 0.525 mL of precipitant, nanoparticles are created, of which the diameter increased sharply to about 185 nm (Table 4).

**Table 4:** Diameter of tin dioxide particles as a function of the amount of precipitant (ammonium hydroxide).

Amount of NH_4_OH (mL)	Diameter (nm)

0.210	8
0.315	12
0.410	12
0.525	185
0.630	185

**Figure 9:**
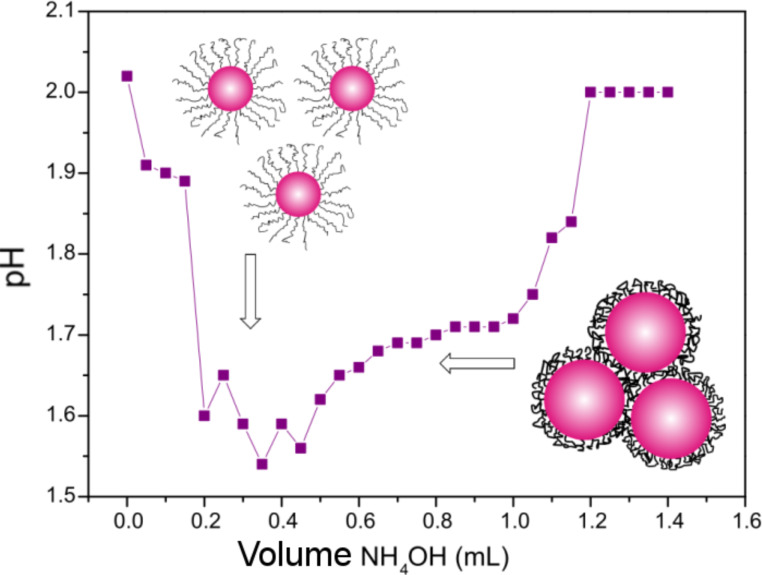
pH change as a function of the amount of precipitating agent (ammonium hydroxide) added.

According to these results, SnO_2_ nanoparticles with the smallest diameters are created when the ratio of non-stoichiometric OH^–^ ions to Sn^4+^ ions is 3:4. This is due to the change in pH of the reaction mixture causing not only the precipitation of tin dioxide particles, but also the change in the structure of the stabilising polymer. The shape of the polymer molecules depends not only on the structure of the polymer chain, but also on the pH of the solution, as shown in Figure 7. According to the literature, with the addition of tin(IV) chloride to a solution of PEI, a series of simultaneous processes occurs: the PEI protonation and formation of tin(IV)-ion complexes with PEI [23]. This complex decomposes under the influence of hydroxide, and tin dioxide nanoparticles are formed in the polymer matrix, as shown in Figure 5. PEI deprotonation occurs simultaneously and its chain begins to roll up. At the appropriate pH and PEI chain length, agglomeration of nanoparticles does not occur (Figure 9).

In order to confirm the hypotheses presented herein, changes in the solution acidity while adding the precipitating agent (ammonium hydroxide) (Figure 9) were observed by DLS to determine the average nanoparticle diameter (Table 4).

The pH of an aqueous solution containing only PEI is 9.66, and after addition of a solution of tin(IV) chloride, it rapidly decreases and reaches the value of 2.02 (Table 5). After the addition of the first portion of ammonium hydroxide, the acidity of the solution increases, and the smallest diameter nanoparticles are precipitated when the pH value is the lowest. In solution, the ratio of OH^–^ ions to Sn^4+^ ions is stoichiometric, then the pH begins to increase and the size of the nanoparticles increases rapidly. These results confirm the hypothesis of the competitive protonation and deprotonation processes of the polymer in the tested solution. However, the balance between these two processes can be effectively controlled with the acidity of the solution, because PEI (which has undergone deprotonation) does not stabilise the nanoparticles.

**Table 5:** pH changes resulting from the stabilising agents and the precursor.

Composition of the solution	pH

H_2_O + PEI	9.66
H_2_O+ PEI + SnCl_4_	2.02
H_2_O + PVP	5.02
H_2_O + PVP + SnCl_4_	1.70

**Figure 10:**
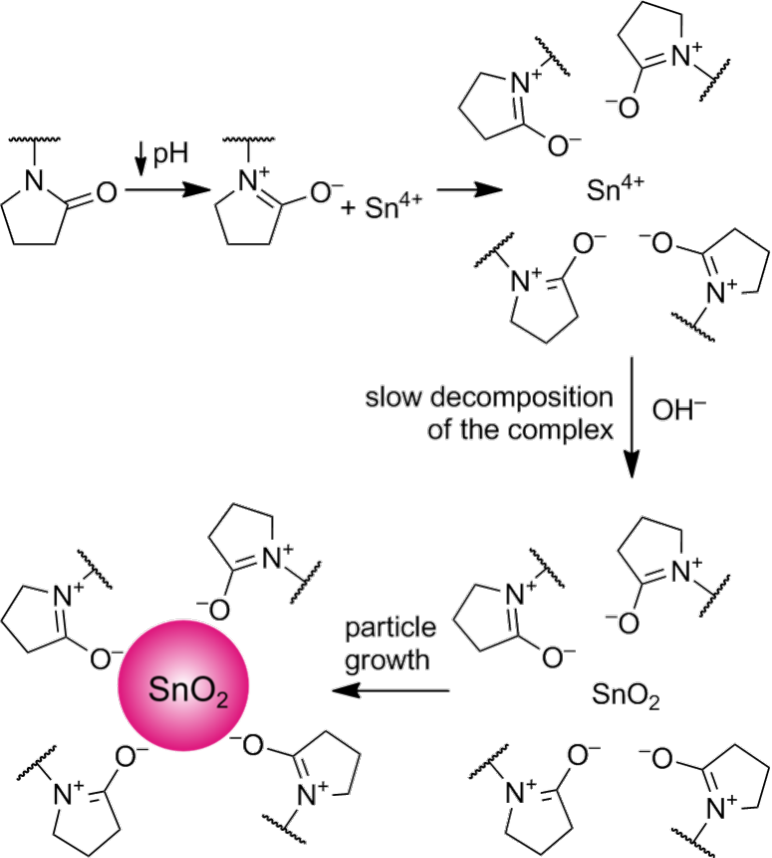
Diagram of the process of SnO_2_ particle formation in the presence of PVP.

In the presence of PVP (the second of the stabilisers used, Table 2 and Table 5), the process happens differently. PVP is a linear, polyelectrolyte polymer which is also highly soluble in water. PVP forms complexes with various compounds and particularly with H-donors, such as carboxylic acids [28]. It is characterised by its high affinity to many chemicals and forms coordination compounds with them due to its strong polar group (vinyl ring) [29]. A PVP chain is more ordered than PEI due to the presence of these rings in the chain. In acidic aqueous solutions, charge transfer between the nitrogen and oxygen atoms of PVP occurs [29]. For this reason, in an acidic medium (pH 1.70, which is a mixture comprising tin(IV) chloride and PVP), a PVP coordination complex is formed with Sn^4+^ ions. Then, upon addition of hydroxide ions into the solution containing PVP and SnCl_4_, two processes occur. The complex is decomposed and tin dioxide nanoparticles are precipitated. According to the literature, the durability of the complex is high. For this reason, the resulting SnO_2_ particles are poorly stabilised because they crystallise quickly. The mechanism of the SnO_2_ particle creation in the presence of PVP is shown in Figure 10. In addition, as a result of the charge transfer between the nitrogen and oxide atoms, the PVP molecule is linear and does not form clusters, and as a result, the nanoparticles are poorly protected against agglomeration.

**Figure 11:**
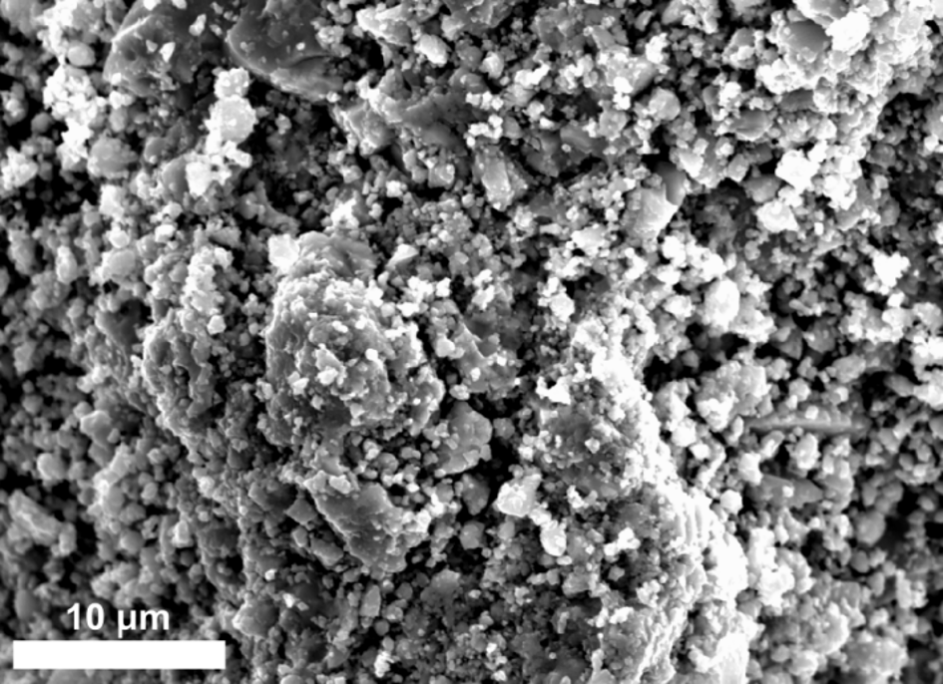
SEM image of SnO_2_ particles synthesized with PVP (*M*_w_ = 58 000).

Due to insufficient protection against agglomeration in the presence of PVP, micrometer-sized particles of SnO_2_ are formed. The SEM image of Figure 11 confirms the results obtained by DLS (Table 2). The SnO_2_ particles have a diameter of about 2 µm after the removal of water and stabilizers.

## Conclusion

This study was focused on the influence of the individual solution components on the size of tin dioxide nanoparticles obtained by a precipitation reaction with ammonium hydroxide. The analysis of the results showed that during the preparation of the tin dioxide nanoparticles (which result from the precipitation reaction), it is necessary to use both a polymer as well as a surfactant. This reduces the surface tension, making it easier to change the structure of the stabilising polymer chain. In addition, not only the concentration of the particular reactants is important, but also their interrelation.

The nanoparticle diameter was dependent not only upon the type of stabilising polymer, but also on the concentration and acidity of the solution, which was varied by addition of a precipitant. As the acidity of the solution changes, a protonation or deprotonation change and straightening of the PEI chain occurs. PEI in acidic solution (at precipitant deficiency) undergoes protonation and its particles have the shape of threads. When the average molecular weight of this polymer is high, it acts as a good stabiliser, and nanoparticles with an average diameter of about 10 nm are formed. In contrast, when the pH increases, PEI particles undergo deprotonation and its concentration increases. Then the chain begins to roll and the chains of the polymer attach to a single nanoparticle along with the intertwined chains surrounding the other nanoparticles. In such a case, agglomerates are formed as was confirmed in the TEM images. This implies that, in addition to the need for a polymeric stabiliser having a relatively long chain, it is necessary to maintain an appropriate pH of the reaction mixture for complete stabilisation.

When using PVP as a stabilising agent, processes are carried out differently. In the PVP chain, ring substitutes are present, therefore, this chain is more orderly than the PEI chain. Due to the nitrogen and oxygen atoms present in the PVP ring, a charge transfer takes place in acidic solution and a coordination complex of PVP with tin ions is formed. Under the influence of the precipitation agent (ammonium hydroxide), the PVP coordination complex with Sn^4+^ ions is decomposed and the tin dioxide nanoparticles are precipitated. Since the durability of the complex is high, the crystallisation and growth of precipitating SnO_2_ particles is faster than their stabilisation. In addition, as a result of the charge transfer between the nitrogen and oxide atoms, the PVP molecule is linear and does not form clusters. As a result, the nanoparticles are poorly protected against agglomeration when PVP is used as a stabiliser.

## Experimental

### Synthesis of SnO_2_ nanoparticles

The synthesis of tin(IV) oxide nanoparticles was carried out in an aqueous solution containing a precursor of tin ions, the polymer and a non-ionic surfactant. Nanoparticles were formed as a result of the precipitation reaction [30]. As the precursor of tin ions, anhydrous tin(IV) chloride from Sigma-Aldrich was used. As the non-ionic surfactant, tert-octylphenoxypolyethoxyethanol (Triton X100) with HLB = 13.5 was used, and high, medium and low molecular weight PEI and PVP from Sigma-Aldrich were used as stabilising agents. From PEI and PVP, aqueous solutions of concentrations 43 g/L and 111 g/L, respectively, were prepared, which corresponded to solutions of 1 mol/L concentration as calculated by the molar mass of the -mer per litre. During the synthesis, an aqueous solution containing tin ions, the polymer and the non-ionic surfactant were mixed and the flocculation agent (ammonium hydroxide) was added in aliquots.

The solution with the standard composition contained 8.24 mL of water, 3 mL of PEI, 6.56 mL Triton (with a concentration of 0.08 mol/L), 0.1 mL of SnCl_4_ (with a concentration of 0.375 mol/L), and 0.21 mL of ammonium hydroxide (with a concentration of 0.0714 mol/L). During the study, the effect of the concentration of the different solutions on the average diameter of the nanoparticles was observed.

To confirm the presence of and determine the diameter of SnO_2_ nanoparticles in the sample, the following methods were used: UV–vis, DLS and XRD. The influence of the concentration of the stabilizing agents was tested by changing only the amount of one component in standard composition at a time. For example to analyse the effect of the amount of surfactant on the diameter of the SnO_2_ particles, only the surfactant concentration was changed, while the amount of other components was held constant.

### Characterisation of SnO_2_ nanoparticles

**UV–vis spectroscopy:** Spectroscopic studies were performed using an Evolution 201 UV–vis spectrophotometer from Thermo Scientific. The studies were performed at 23 °C in quartz cuvettes.

**Dynamic light scattering (DLS):** The diameter distribution of the resulting nanoparticles was determined by DLS. The studies were performed using a Nicomp 380ZLS Particle Sizing System with an excitation wavelength of 532 nm at 50 mW. The frequency of the photon counting was fixed at about 200 kHz, and each measurement was performed for 3 min. A poly(methyl methacrylate) (PMMA) cuvette with dimensions of 40 × 10 × 10 mm was used for the measurements. The calculation of the hydrodynamic diameter was performed on the basis of the Stokes–Einstein equation, assuming a measurement temperature of 298 K and a viscosity of the continuous phase (water) of 0.891 mPa∙s.

**Transmission electron microscopy (TEM):** Microstructure studies of the resulting tin oxide particles were made using an EM900 TEM (Zeiss, Germany). The samples were prepared by depositing drops of a suspension of SnO_2_ nanoparticles onto a copper mesh (200 mesh) covered with carbon and coated with Formvar resin. Data analysis and processing was performed using AxioVision 4.8 software (Zeiss) and ImageJ 1.42q. The study was performed at ambient temperature, 25 °C.

**X-ray diffraction (XRD):** Diffraction studies were performed using a Philips materials research diffractometer. The samples were dried and heated at 450 °C for 15 min.

**Scanning electron microscope (SEM):** Microstructure studies of the tin oxide particles were made using an LEO 435 VP scanning electron microscope (SEM) (Zeiss, Germany). The samples were dried and heated at 600 °C for 30 min. Before the measurement, a gold layer was deposited on the sample using a coater (Edwards–Pirani 501).
